# A deep learning image-based intrinsic molecular subtype classifier of breast tumors reveals tumor heterogeneity that may affect survival

**DOI:** 10.1186/s13058-020-1248-3

**Published:** 2020-01-28

**Authors:** Mustafa I. Jaber, Bing Song, Clive Taylor, Charles J. Vaske, Stephen C. Benz, Shahrooz Rabizadeh, Patrick Soon-Shiong, Christopher W. Szeto

**Affiliations:** 1 NantOmics LLC, 9920 Jefferson Blvd., Culver City, CA 90232 USA; 2 ImmunityBio, 9920 Jefferson Blvd., Culver City, CA 90232 USA; 3 Department of Pathology, Keck School of Medicine, University of Southern California, HMR 2011 Zonal Ave., Health Sciences Campus, Los Angeles, CA 90033 USA; 4 ImmunityBio, 2901 Mission St. Ext., Santa Cruz, CA 95066 USA

**Keywords:** Breast cancer, Intrinsic molecular subtype (IMS), Whole-slide imaging (WSI), Deep learning algorithm

## Abstract

**Background:**

Breast cancer intrinsic molecular subtype (IMS) as classified by the expression-based PAM50 assay is considered a strong prognostic feature, even when controlled for by standard clinicopathological features such as age, grade, and nodal status, yet the molecular testing required to elucidate these subtypes is not routinely performed. Furthermore, when such bulk assays as RNA sequencing are performed, intratumoral heterogeneity that may affect prognosis and therapeutic decision-making can be missed.

**Methods:**

As a more facile and readily available method for determining IMS in breast cancer, we developed a deep learning approach for approximating PAM50 intrinsic subtyping using only whole-slide images of H&E-stained breast biopsy tissue sections. This algorithm was trained on images from 443 tumors that had previously undergone PAM50 subtyping to classify small patches of the images into four major molecular subtypes—Basal-like, HER2-enriched, Luminal A, and Luminal B—as well as Basal vs. non-Basal. The algorithm was subsequently used for subtype classification of a held-out set of 222 tumors.

**Results:**

This deep learning image-based classifier correctly subtyped the majority of samples in the held-out set of tumors. However, in many cases, significant heterogeneity was observed in assigned subtypes across patches from within a single whole-slide image. We performed further analysis of heterogeneity, focusing on contrasting Luminal A and Basal-like subtypes because classifications from our deep learning algorithm—similar to PAM50—are associated with significant differences in survival between these two subtypes. Patients with tumors classified as heterogeneous were found to have survival intermediate between Luminal A and Basal patients, as well as more varied levels of hormone receptor expression patterns.

**Conclusions:**

Here, we present a method for minimizing manual work required to identify cancer-rich patches among all multiscale patches in H&E-stained WSIs that can be generalized to any indication. These results suggest that advanced deep machine learning methods that use only routinely collected whole-slide images can approximate RNA-seq-based molecular tests such as PAM50 and, importantly, may increase detection of heterogeneous tumors that may require more detailed subtype analysis.

## Background

Immunohistochemistry (IHC) or in situ hybridization (ISH) assays are well-established methods used to distinguish subtypes in breast cancer (BC) based on hormone receptor statuses [1]. Increasingly, RNA-based signature assays such as MammaPrint, Oncotype DX, and Predictor Analysis of Microarray 50 (PAM50) [2] are being employed as supplementary prognostic indicators due to studies demonstrating more significant differential survival between identified subtypes when compared to standard clinicopathological factors [3–5]. In particular, PAM50 intrinsic molecular subtyping, as part of the NanoString Prosigna [6] and the Agendia BluePrint panel [7], is becoming more widely used in early-stage breast cancers to determine the likelihood of responding to chemotherapy. The PAM50-defined intrinsic molecular subtype (IMS) classifications include Luminal A (LumA), Luminal B (LumB), HER2-enriched (HER2), Basal-like (Basal), and Normal-like (Normal); while there is some correlation between receptor status and IMS, the latter is determined by consideration of gene expression beyond receptors. The molecular signature-based tests are not, however, as ubiquitously employed as IHC-based receptor subtyping in part due to their high cost, extended processing times, and requirement for appropriate tissue samples. Thus, a method for tumor classification beyond receptor subtyping that approximates PAM50 subtyping that is practical, cost-effective, and utilizes readily available samples could be of great utility.

Unlike the samples needed for molecular signature assays, hematoxylin and eosin (H&E)-stained biopsy slides are routinely collected during pathological examination, and are often digitally recorded as whole-slide images (WSIs) [8].

Machine learning approaches can extract knowledge from WSIs beyond that of which a human is capable, as evidenced by the many computer-assisted diagnosis (CAD) software solutions created to augment pathological inspection workflows [8]. It has been demonstrated previously that even genetic subtyping can be approximated using WSIs as input to relatively simple machine learning algorithms [9].

Deep learning methods are an emerging set of influential machine learning technologies well suited to these image-based classification tasks [10]. Recent advances in both computational power and convolutional network architectures have greatly increased the applicability of these techniques for several new domains in biology including omics analysis, biomedical signal processing, and biomedical imaging [11]. Specifically, deep learning has been applied to greatly improving detection of regions of interest in BC WSIs [12] and impressive progress has been made in application of deep learning to BC diagnosis from images [13–15].

Of particular interest in WSI analysis is the use of multiscale patch representations that allow concurrent use of high-zoom patches that capture cellular level information with lower-zoom patches that capture global interdependence of tissue structures [16–18]. Bejnordi et al. used multiscale patch representation of WSIs to build highly accurate context-aware stacked convolutional neural networks (CNN) for distinguishing between invasive ductal carcinomas (IDC) and benign ductal carcinoma in situ (DCIS) [19]. Similarly, Liu et al. used this same approach to accurately detect whether biopsy samples from nearby lymph node tissue were positive for metastases [20].

While use of multiscale patch representations may increase performance in WSI-based classification tasks, the computational complexity of training on all possible multiscale patches from gigapixel WSIs is substantial. As such, previous studies have employed strategies that limit the analyzed patches to a subset of the total image. For example, in a study of subtypes in BC, Verma et al. used a minimum filter on the blue–yellow channel at × 20 magnification to select patches rich in epithelial cells [21]. Similarly, in a study of non-small cell lung cancer WSIs, Yu et al. successfully used only the top ten cell-dense 1000 × 1000 pixel (250 × 250 μm) patches at × 40 magnification. However, both of these strategies leveraged tissue-specific knowledge of cell morphology in their respective indications [22]. Generalizable methods for focusing on information-rich image patches are an area of ongoing research.

Here, we present a method for minimizing manual work required to identify cancer-rich patches among all multiscale patches in H&E-stained WSIs that can be generalized to any indication. A minimal number of such cancer-rich WSI patches were then used to classify tumors into IMS, i.e., PAM50 WSI-based subtypes.

Similarly to the method presented here, Couture et al. [23] recently applied deep learning to image analysis to predict BC grade, ER status, and both histologic and intrinsic subtype when modeled as binary classifiers (i.e., Basal-like vs. non-Basal-like) and achieved > 75% accuracy, supporting development of such classifiers. They used 1 mm cores from pathologist-marked areas (1–4 per WSI) for tissue microarray (TMA) construction, and the authors noted that cores taken from a single slide often classify as different intrinsic subtypes, which may be evidence of heterogeneity. However, characterizing the extent of intrinsic subtype heterogeneity from TMAs would be extremely difficult even with multiple small cores from a single WSI.

One distinct advantage of the patch-based WSI-based IMS classifier described here is retention of the ability to observe intratumoral heterogeneity directly without resorting to numerical deconvolution methods. We leveraged this patch-based system to identify tumors presenting at least two molecular subtypes within the same tissue section, and support these cases as mixed populations using independent data including overall survival. Others have previously used image-based measures of heterogeneity as prognostic biomarkers [24], but to our knowledge, this is the first study of prognostic intrinsic subtype heterogeneity identified in diagnostic WSIs.

## Methods

### Constructing multiscale patch representations

All diagnostic WSIs of H&E-stained sections from formalin-fixed paraffin-embedded (FFPE) blocks collected from 1097 patients with invasive BC were obtained from The Cancer Genome Atlas (TCGA) data sources [25], resulting in a collection of 1142 diagnostic WSIs. WSIs were tiled into 1600 × 1600 pixel (800 × 800 μm) patches at the × 20 zoom level. All 1600 × 1600 pixel patches were filtered for a minimum color variance to eliminate empty (background) patches from further processing. Each 1600 × 1600 pixel 20× patch was converted into 400 × 400 pixel patches at × 5, × 10, and × 20 magnification scales centered on the same point by down-sampling and cropping to the center 400 × 400 pixels. Next, a deep CNN was used to transform 2D color patches into classifiable 1D descriptive vectors as follows: 2D patches were input into a version of the *Inception v3* network (Google) [26] pre-trained on the ImageNet database of images to classify a wide variety of objects. The representations at the final layer of the network (the logits layer) were then extracted. This process maps each 400 × 400 pixel color patch into highly descriptive vectors with 2048 dimensions at each zoom level. Principal component analysis (PCA) was used to reduce dimensions while retaining > 96% variance. Finally, vectors for all three zoom levels were concatenated into one multiscale patch representation.

### Enriching for cancer patches

For training, 238,728 multiscale patch representations were randomly selected. These representations were grouped using *k*-means clustering; the number of clusters was determined empirically. Clusters with sufficient cellularity were investigated further. A pathologist evaluated 336 representations for tumor content. The clusters were assessed for cancer enrichment by observing the percentage of patches within said clusters that were also positive for tumor content. For each WSI, up to 80 patches that fell within the cancer-rich clusters were used for further analysis. If a WSI contained more than 80 cancer-rich patches, only 80 were selected at random.

### PAM50 classification

Both PAM50 expression-based molecular subtyping and survival data were available for 789 out of 1097 BC patients used for our WSI-based IMS classifier development; the subtypes comprised 50.4% LumA, 21.7% LumB, 16.9% Basal, 8.1% HER2, and 2.9% Normal.

Because a large number of example patches are generated from each patient, ensuring that the majority of patches from each training patient are of one subtype is important. In order to remove likely heterogeneous patients from the training pool to allow training utilizing only the most strictly defined subtype, patients were assessed for how closely their gene expression as determined by RNAseq associated with other patients from their assigned subtype. Gene expression values (as RSEM transcripts per million values) for the 50 PAM50 genes were obtained from TCGA sources (https://gdac.broadinstitute.org). These expression profiles were used to cluster all 789 patients in the PCA-space, an unsupervised analytical method for gene expression data that provides a picture of the overall distribution of the analyzed dataset [27]. Patients were deemed low-confidence if the Euclidean distance to their assigned subtype centroid was > 33% larger than the distance to the nearest subtype centroid (Additional file 1: Figure S1). A total of 104 patients were assigned the low-confidence (i.e., likely heterogenous) label; elimination of such cases for training is a method used by others [23].

The normal-like subtype (tumor tissue with gene expression similar to normal breast tissue) was deemed insufficiently represented for multiclass classification (*n* = 23) and dropped from training, resulting in a 4-way classification task. The 766 non-normal-like patients were split into training (*n* = 443; 58%) and validation (*n* = 323; 42%) datasets. All 101 non-normal-like low-confidence patients were assigned to the validation set. Patients in the training dataset were further split into 5 pairs of training and testing datasets (i.e., fivefold cross-validation). Within each fold, 60 multiscale cancer-enriched patches were selected per training WSI and used to train a multiclass one-vs-rest support vector machine (SVM) with radial basis function (RBF) kernel. Trained models were used to classify 80 multiscale cancer-enriched patches from each testing WSI, then aggregated via majority voting to classify at the tumor level. In the few cases where a patient had multiple diagnostic slides, a voting mechanism was used to assign the patient’s overall IMS label. A final multiclass one-vs-rest SVM with RBF kernel classifier was trained on all 443 training WSIs and analyzed for subtyping accuracy in the validation set of unseen patients.

### Detecting subtype heterogeneity

Analysis of heterogeneity focused on detecting the two subtypes with most dissimilar survival characteristics (LumA and Basal). To label patients as LumA by image (LumA_IMG_), a threshold for the minimum percentage of patches classified as LumA was determined using Youden’s analysis [28] in the training set as follows: patient tissue was assigned “LumA” or “not LumA” labels according to PAM50 molecular subtyping, then all percentages of LumA patches were considered and the threshold that maximizes the true positive rate (TPR) to false positive rate (FPR) ratio was selected. A threshold for calling WSI Basal by image (Basal_IMG_) was found similarly. Patients were categorized as heterogeneous (HET), LumA_IMG_, and Basal_IMG_ using these pre-trained thresholds. Heterogeneity was supported by analysis of Mann-Whitney *U* tests of HR expression and Kaplan-Meier survival curves with Cox proportional hazard analysis.

### Binary classification: Basal vs. non-Basal

Based on the findings from the above initial establishment of the classifier, we sought to increase its prognostic utility by re-defining subtyping as Basal or non-Basal (HER2, LumA, LumB, and Normal). To do this, we used the top 60 multiscale patches from 582 WSIs (92 Basal and 490 non-Basal) to train a binary linear SVM classifier with *C* = 1.0. The training data had 34,745 multiscale patches (15.84% Basal and 84.16% non-Basal) and resulted in patch-level train accuracy of 90.58% (with sensitivity = 64.72% and specificity = 95.44%).

In addition, to improve the sensitivity of the Basal vs. non-Basal classifier, we employed a class balance technique of training patches to train another “balanced” linear SVM (*C* = 1.0) classifier. That is, a set of 5.5K multiscale patches were randomly selected from the Basal and non-Basal classes.

## Results

### Image-based IMS classifier pipeline design

**Fig. 1:**
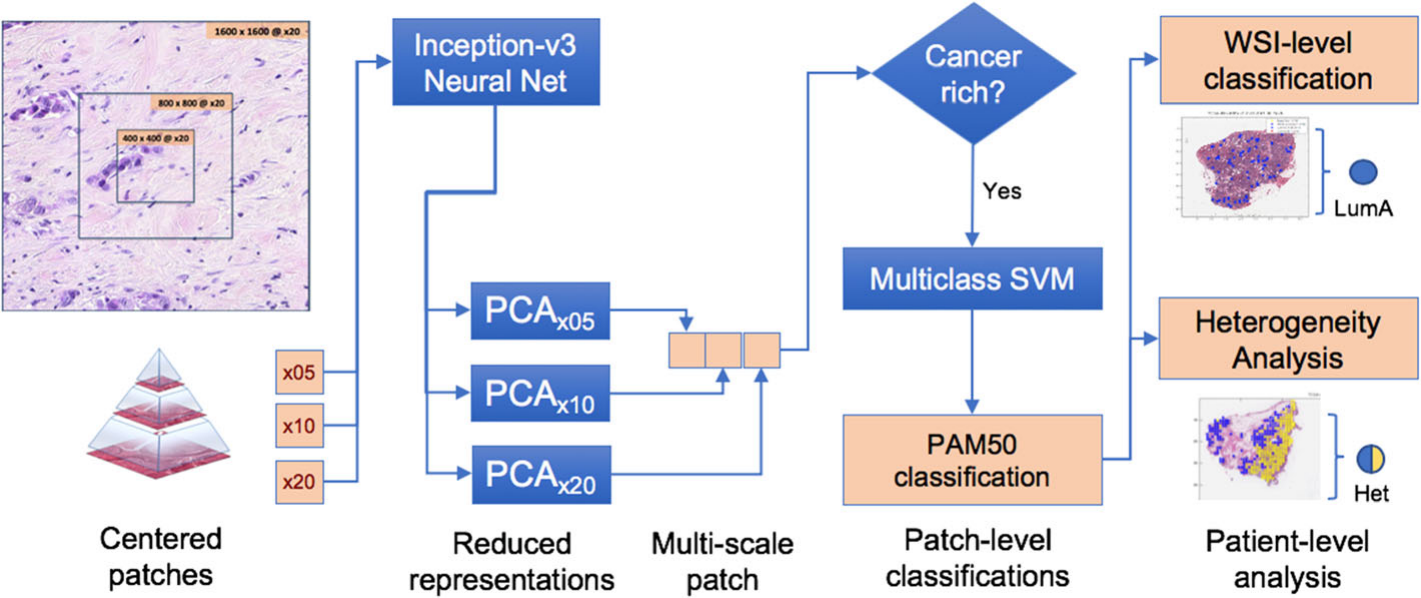
Proposed WSI-based IMS classifier and heterogeneity detection system. WSIs are broken into multiscale 400px × 400px patches and converted to descriptive tensors using the Inception v3 neural net architecture. A subset of cancer-enriched patches is selected to summarize WSI tumor content. Each patch is assigned a subtype in a 4-way classifier (Basal-like, HER2-enriched, Luminal A, and Luminal B). WSI-based subtype classifications can be made by employing a voting mechanism upon the patch-based results. Heterogeneity analysis is further performed on WSIs displaying significant concurrent Basal-like and Luminal A image-based predictions

The proposed system for classifying H&E-stained diagnostic WSIs into intrinsic molecular subtypes is shown in Fig. 1. A fixed-size multiscale patch-based approach was selected to allow analysis of regions as well as capture micro- and macroscopic characteristics simultaneously. The *Inception v3* logits representation of color patches was used to convert color representations into descriptor vectors because it is particularly well suited to multiscale patch representation. A system for filtering analyzed locations to cancer-enriched locations (as opposed to extracellular matrix or adjacent normal tissue) was employed to reduce computational complexity and ensure hygienic input. A multiclass SVM classification algorithm was trained due to superior performance on large datasets.

### Multiscale patch representations

The average for the 1142 WSIs from 1097 BC patients was 5465 × 11,641 pixels (10.93 × 23.28 mm) at the × 5 magnification level, resulting in 2,709,065 total analysis locations. After applying color filtering to remove non-tissue areas, 1,985,745 locations remained. Each location was down-sampled from the × 20 zoom level to represent × 20, × 10, and × 5 zoom levels centered on the same location, resulting in 5,957,235,400 × 400 pixel color patches. These two-dimensional color patches were converted to vectors of length 2048 by the *Inception v3* logits layer. PCA was applied to 5×, 10×, and 20× vectors independently, and various levels of dimensionality reduction were explored (Additional file 1: Table S1). A length of 768 components was found to retain > 96% variance in each zoom level. After converting images to multiscale patch representations, the total dataset size is a matrix of 1,985,745 locations × 2304 features.

### Cancer enrichment

From the total of 238,728 multiscale patch representations randomly selected for defining cancer-enriched centroids, we identified 24 clusters using *k*-means clustering. Fourteen of the 24 clusters were sufficiently populated with cellular structures for further analysis. A pathologist annotated 24 patches from each cluster (336 total) to determine whether or not the patch contained tumor tissue (Additional file 1: Table S2). Five leading clusters had mostly cancer-rich samples (> 80% of patches are cancer-rich). Additional file 1: Figure S2 presents examples of these five clusters and their prevalence in the patch-level population.

### WSI-based IMS classification

Table 1 summarizes the accuracy of subtype classifications at the patch, WSI, and patient level in held-out test samples in fivefold cross-validation of the training samples. On average, 354 WSIs were used to train and 94 were used to test accuracy. Within the held-out test WSIs, individual patches were classified less accurately than when aggregated into a single WSI-level classification (58.6% vs. 66.1% correct). When multiple diagnostic WSIs are available for a given patient, aggregating across slides may also increase accuracy (66.1% vs. 67.3% correct).

**Table 1:** Molecular subtyping accuracy across folds. Sample size and performance statistics within the held-out test set across fivefold cross-validation

	No. of patches	No. of WSIs	No. of patients	Patch-level accuracy (%)	WSI-level accuracy (%)	Patient-level accuracy (%)
Fold1	7505	95	92	60.47	70.53	71.74
Fold2	7501	94	89	56.97	67.02	67.42
Fold3	7581	95	88	57	67.37	69.32
Fold4	7564	95	86	59.56	65.26	65.12
Fold5	7420	93	88	59.1	60.22	62.5
Average	7514.2	94.4	88.6	58.62	66.1	67.27

**Fig. 2:**
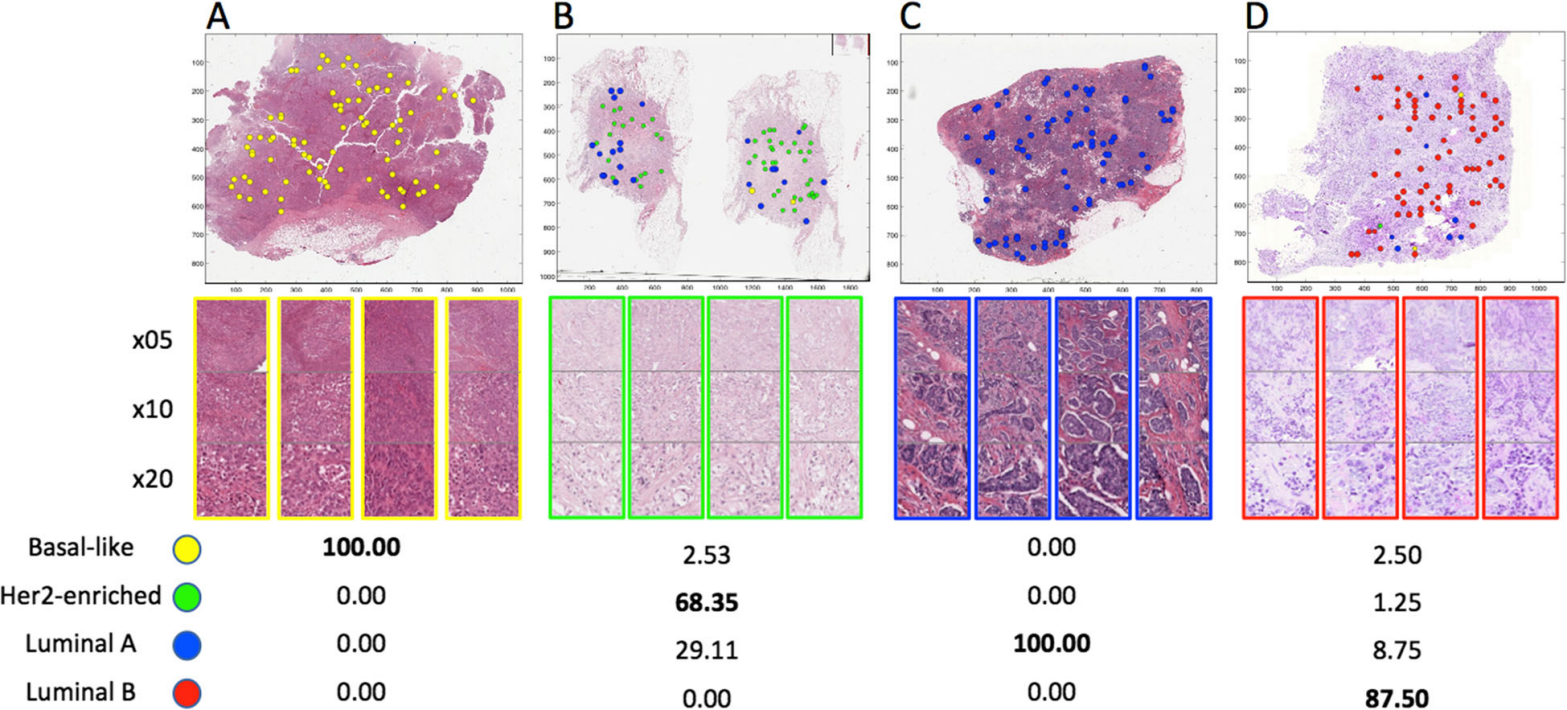
Subtyping cancer-enriched multiscale patches. Four examples of patch-level subtype classifications: **a** Basal-like, **b** HER2-enriched, **c** Luminal A, and **d** Luminal B. Below each WSI are 4 example multiscale patch representations from the 80 selected. The bottom table shows the percentages for each predicted subtype within the selected cancer-rich multiscale patches

Table 2 shows performance in two validation sets: one unselected group of 222 patients, and a second group containing 101 patients with low-confidence IMS classifications. Within the group of unselected patients, tumor subtype classification performance was similar to the cross-validated setting (65.9% vs. 67.3% correct). The main sources of error were misclassification of LumA tumors as LumB and of Basal into other subtypes. Within the low-confidence patients, overall subtyping accuracy was much lower (56.7% correct), potentially due to subtype heterogeneity. It should be noted that the automatic masking system used is not capable of determining which patches are non-cancer rich within the cancer-rich clusters; thus, there is possibility that in some cases, heterogeneity comes from non-cancer patches. Figure 2 shows patch-level subtype classification results on four WSI examples.

**Table 2:** Molecular subtyping error and accuracy in two test settings. Confusion matrices between true labels (RNA-seq-based IMS in columns) and predicted labels (WSI-based IMS in rows) at the patient-level for unselected (left) and low-confidence (right) by RNA-seq-based classification

	Unselected test patients (*N* = 222)				Low-confidence test patients (*N* = 101)			
	Basal-like	HER2-enriched	Luminal A	Luminal B	Basal-like	HER2-enriched	Luminal A	Luminal B
Basal-like	13	0.9	0.9	0	0.96	0	0	0
HER2-enriched	4.48	1.79	2.24	0.45	0.96	0	0	0
Luminal A	1.35	1.79	46.19	2.69	4.81	1.92	43.27	1.92
Luminal B	6.73	0.9	11.66	4.93	6.73	2.88	24.04	12.5
	Patient-level accuracy 65.92%				Patient-level accuracy 56.73%

### The WSI-based IMS classifier identifies LumA, Basal, and HET tumors

**Fig. 3:**
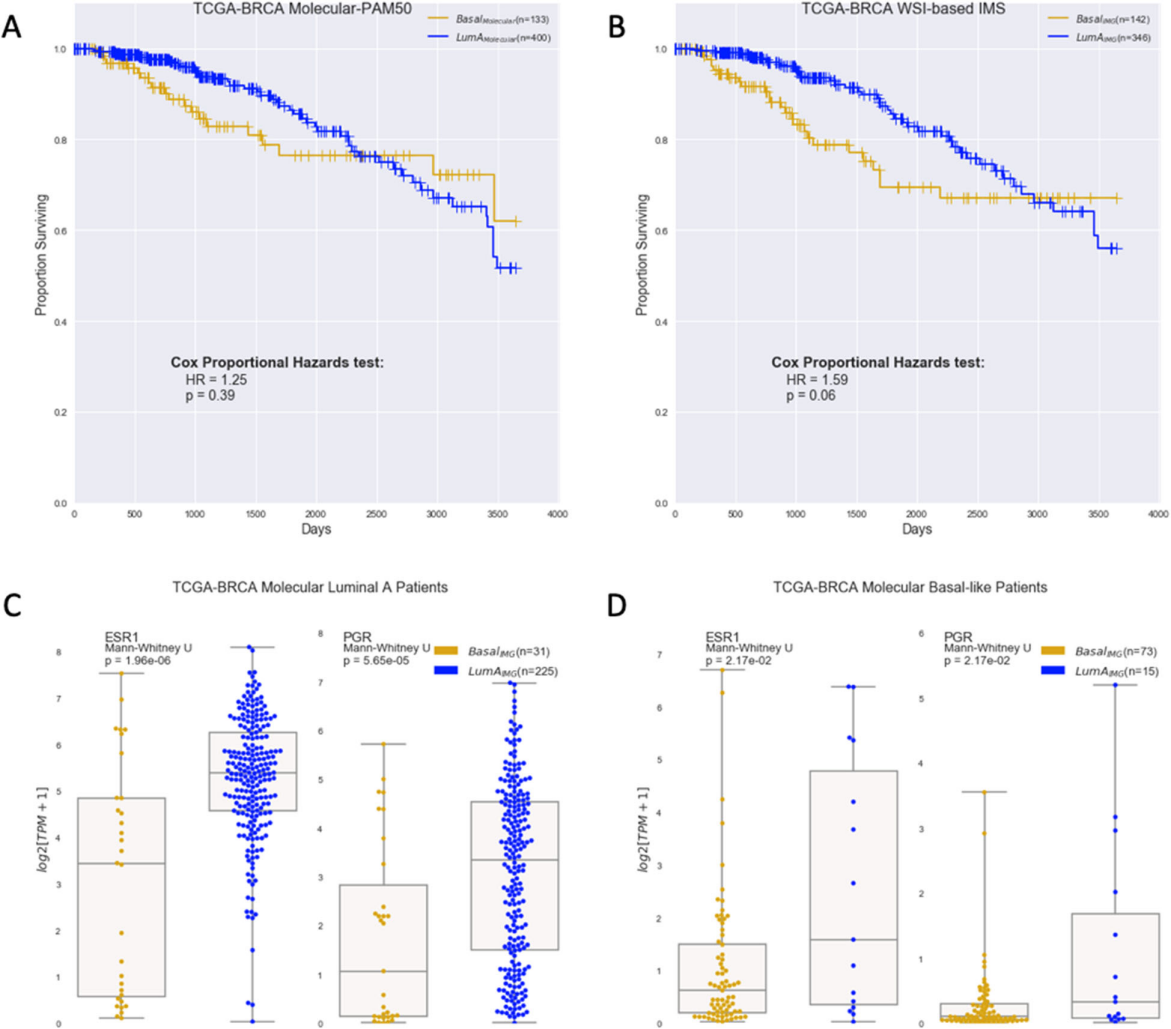
WSI-based IMS vs. RNA-seq-based molecular PAM50. **a** Kaplan-Meier curves for Luminal A and Basal-like based on molecular PAM50 calls with HR = 1.25 and log-rank tests *p* = 0.39 (*n* = 533). **b** Kaplan-Meier curves for Luminal A and Basal-like based on WSI-IMS calls with HR = 1.59 and log-rank tests *p* = 0.06 (*n* = 488). **c** All the cases analyzed were molecularly classified as LumA, but the WSI-based system classified some of these (*n* = 31) as Basal (yellow); the expression levels of ESR1 and PGR for cases WSI-subtyped as Basal were lower compared to confirmed LumA (blue). **d** Conversely, the receptor levels of molecularly subtyped Basal cases WSI-subtyped to be LumA (*n* = 15) are higher than confirmed Basal cases

Of the five molecularly based classifications for all BC patients in TCGA, the two major subtypes with good survival separation are LumA and Basal as shown in the Kaplan-Meier survival curves in Fig. 3a; the hazard ratio (HR) = 1.25 and *p* = 0.39 characterize the difference between the two curves. WSI-based IMS reveals four subtypes—LumA, LumB, HER2, and Basal—with LumA and Basal also showing good survival-curve separation, as shown in Fig. 3b (HR = 1.59; *p* = 0.06). This side-by-side comparison reveals good correlation of the WSI- to molecularly based classification and survival. Figure 3b also reveals that the WSI-based IMS classifier provides more distinctive differential prognosis between LumA and Basal subtypes than molecular IMS in the TCGA dataset.

The WSI-based classifier identified a majority (255/400) of molecularly subtyped LumA patients as LumA; the rest (175) were classified as Basal (31), HER2, or LumB. The discrepancy is not necessarily an error of WSI-based classification because other factors such as the levels of two key breast-related receptors—the estrogen receptor alpha (ERα/*ESR1*) and progesterone receptor (PR/*PGR*)—support the accuracy of the WSI-IMS call. For example, the WSI-IMS Basal group expresses lower levels of ESR1 and PGR than the WSI-IMS LumA group (Fig. 3c). Results were similar for the TCGA molecularly subtyped Basal-like cohort (133) where our image-based algorithm identified a majority (73) as Basal-like patients, the rest (60) as HER2, LumA (15), or LumB. The LumA_IMG_ group (molecularly identified as Basal, but identified as LumA_IMG_ by proposed system, which has 15 patients) expresses higher levels of key hormone receptors when compared to Basal_IMG_ group (Fig. 3d).

In Additional file 1: Figure S3, analyses performed for Fig. 3 above were repeated, but using the test data only for unselected and low-confidence (patients in Table 2). Thus, fewer patients as compared to Fig. 3 above are represented. The Kaplan-Meier curves for LumA and Basal based on molecular PAM50 calls have an HR = 1.27 and log-rank tests of *p* = 0.60 (Additional file 1: Figure S3a); based on WSI-IMS calls, they are HR = 1.66 and log-rank tests *p* = 0.11 (Additional file 1: Figure S3b). The receptor expression results are similar to those in Fig. 3.

To define LumA_IMG_ and Basal_IMG_ patients in Fig. 3b, thresholds that maximized agreement between patch-based classifications and molecular-based classifications were identified using Youden’s analysis (Additional file 1: Figure S4). A threshold of at least 63.7% of patches classifying as LumA was found to maximize agreement between molecular-based LumA and IMG-based LumA classification, with a true positive rate (TPR) of 0.80 and false positive rate (FPR) of 0.15. At this threshold, 346 patients were classified as LumA_IMG_ by the WSI-based IMS algorithm. Similarly, a threshold of at least 40.5% patches classifying as Basal-like maximized agreement with molecular Basal-like classification, with TPR of 0.81 and FPR of 0.14. This resulted in assigning 142 patients as Basal_IMG_.

**Fig. 4:**
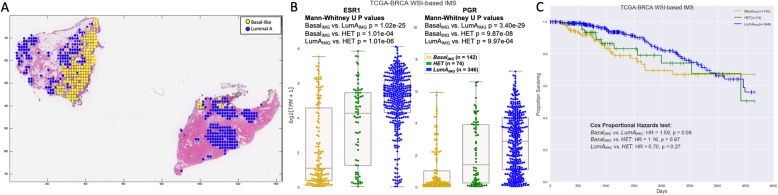
Evidence for heterogeneity. **a** An example of a HET WSI with markup on patches predicted as Basal-like and LumA. **b** Expression levels of key hormone receptors ESR1 and PGR in the three settings. Mann-Whitney *U p* values of being drawn from the same distribution are reported for each pair of settings. Inputs are Basal_IMG_, HET, and LumA_IMG_ cohorts as defined by the WSI-based IMS system. **c** Kaplan-Meier curves for Basal_IMG_, HET, and LumA_IMG_ cohorts show HET survival to be intermediate between the other two. Cox proportional hazard test is included

Furthermore, 74 tumor tissue samples with > 33% of patches classified as Basal and > 33% of patches classified as LumA were considered possibly heterogeneous (HET) samples. Visual evidence for heterogeneity is shown in Fig. 4a where a Basal patient based on molecular PAM50 was identified as HET by the WSI-based IMS. The diagnostic H&E WSI showed subpopulations of both LumA and Basal patches.

HET tumors detected by the proposed WSI-based IMS system are intermediate between LumA_IMG_ and Basal_IMG_ populations based on hormone (Fig. 4b) receptors. Basal_IMG_ and LumA_IMG_ tumor distributions are separated based on Mann-Whitney *U* test with *p* = 1.02 × 10^−25^ in ESR1 and *p* = 3.40 × 10^−29^ in PGR. Furthermore, Fig. 4c shows that while LumA_IMG_ and Basal_IMG_ have differential survival characteristics, the survival of patients with HET tumors is not significantly distinct from either subtype so can be inferred to be intermediate between LumA_IMG_ and Basal_IMG_.

### Application of the Basal/non-Basal binary classifier

The binary classifier was utilized to generate WSI-level results for validation WSIs. The accuracy of patch-level train performance for the binary classifier is shown in Additional file 1: Table S3.

This validation set included 258 WSIs (44 Basal and 214 non-Basal). The top 80 multiscale patches from validation WSIs gave a ROC AUC of 0.8259 and accuracy of 86.82% (sensitivity = 38.64% and specificity = 96.73%). Notice that this accuracy was achieved with a percentage of 15.84% Basal patches in the training set.

The balanced Basal vs. non-Basal classifier improved the overall performance when applied to the validation set, achieving a WSI-level ROC AUC of 0.8607 with accuracy of 87.21% (sensitivity = 68.18% and specificity = 91.12%). The patch-level train performance of this balanced Basal/non-Basal classifier is shown in Additional file 1: Table S4. The overall performance of the Basal vs. non-Basal and the balanced Basal vs. non-Basal classifiers is shown in Additional file 1: Table S5.

## Discussion

Presented here is a system for breast cancer molecular subtype classification using deep learning patch representations of H&E-stained WSIs. Conventionally, such classification is only accomplished using gene expression signatures such as those generated by PAM50; however, the proposed WSI-based IMS classifier methodology presented herein shows promising performance with overall concordance with molecular-based classification of 65.92% and the ability to detect the most aggressive subtype, Basal, with 87% accuracy. Absence of concordance does not necessarily mean the tumor is inaccurately subtyped, as our results on key receptor expression suggest. Rather, application of our methodology may prompt further investigation of subtype accuracy as based on PAM50.

To increase veracity of training examples, we eliminated low-confidence samples; however, even with purified training labels, many of the expression-based characteristics defined by PAM50 genes may not result in visually (image) discernable features [29], leading in part to the observed reduction in concordance between our image-based classifier and expression-based PAM50 subtyping. Specifically, our analysis shows that this WSI-based IMS classifier is highly sensitive to expression of key cell-surface receptors ERα/*ESR1* and PR/*PGR* (see Fig. 3c, Fig. 3d, and Additional file 1: Figure S4). Despite some disparity in subtyping results between expression-based PAM50 subtyping and this image-based analysis that utilizes morphological characteristics, the WSI-based IMS classifier is not inferior to PAM50 in prognostic capability: in fact, in this cohort, the image-based classifier is more prognostic for differential survival between LumA and Basal patients than molecular PAM50 subtyping.

Intratumoral heterogeneity, common in breast tumors—especially in triple-negative breast cancer [30]—may play a role in reducing concordance between our WSI-based IMS classifier and expression-based subtyping. The methodology presented here summarizes patches into a patient-level classification by majority area, whereas expression profiles are summaries based on total transcript counts. As such, concordance of the deep learning classifier with expression-based subtyping may be improved in the future by increasing weight given to cell-dense or transcriptionally overactive patches.

Many tumor heterogeneity models exist, such as cancer stem cells (CSCs) and the clonal evolution model; recently developed lineage-tracing and cell-ablation methods have furthered understanding of the role of the former in cancer [31]. Figure 4a shows that tumor heterogeneity can occur on a small (~ 100 μm) or large (~ 10 mm) scale; the solid tumor heterogeneity model must take this spatial information into account.

Because of its sensitivity to subclonal diversity, our WSI-based IMS classifier may have novel application as a method for detecting intratumoral heterogeneity. Inspection of tumor biopsy tissues that were misclassified revealed patterns of discordant subtypes at the patch level. Further evidence that these tumors are in fact heterogeneous populations was found in hormone-receptor expression levels and survival characteristics. Specifically, patients with tumors that were classified as LumA subtype but had Basal subclones have poorer survival compared to those with homogeneous LumA tumors. The specific regions identified by this classifier could be further confirmed as molecularly distinct by laser microdissection followed with separate molecular characterization of subclones.

While survival differences between HET and LumA or Basal were not significant, the trend of the HET group having intermediate survival is complementary to image- and expression-based evidence for heterogeneity. One limitation of the TCGA BC cohort is the higher proportion of prospective samples resulting in relatively short follow-up times, which reduces the number of events available to power the Kaplan-Meier analysis. Nonetheless, the intermediate survival of the HET group supports the merit of further studies on the effects of tumor heterogeneity as revealed by the WSI-based method here on survival.

## Conclusions

The ability of the WSI-based IMS classifier to identify heterogeneity in cancer cell populations from diagnostic H&E images has significant prognostic implications. Furthermore, the classifier described herein provides more subtyping information than receptor status alone as determined by IHC or ISH. With continued development of the system to increase accuracy, given the availability of WSIs and cost-effectiveness of the methodology, its application to standard prognostic procedures may be accelerated.

## Supplementary information

**Additional file 1: Figure S1.** Identifying low-confidence PAM50 labels. PCA-plot showing clustering of patient samples using PAM50 genes. Subtype centroids are marked in dark circles, with lines to each patient assigned to those subtypes. Euclidean distance in this space was used to identify 104 patients that cluster significantly closer to a non-assigned centroid. **Figure S2.** Exemplary cancer-enriched multiscale patches. A total of 238,728 multiscale patches were clustered into 24 groups by *k*-means clustering and 336 representatives were selected for pathologist interpretation. Shown here are multiple zoom levels for the patches from the five most cancer-rich cluster centroids, as defined by pathologist inspection. Below each example patch are the group proportions and the percentage of patches containing cancer tissue. **Figure S3.** WSI-based IMS vs. RNA-seq-based PAM50 using test data only for unselected and low-confidence samples. WSI-based IMS vs. RNA-seq-based molecular PAM50 on test patients in Table 2 (unselected & low-confidence). **a** Kaplan-Meier curves for Luminal A and Basal-like based on molecular PAM50 calls with HR = 1.27 and log-rank tests *P* = 0.60. **b** Kaplan-Meier curves for Luminal A and Basal-like based on WSI IMS calls with HR = 1.66 and log-rank tests *P* = 0.11. In **c**, all the cases analyzed were molecularly classified as LumA, but the WSI-based system classified some of these as Basal (yellow); the expression levels of ESR1 and PGR for cases WSI-subtyped as either Basal or LumA (blue) are shown. **d** Similarly, the receptor levels of molecularly-subtyped Basal cases WSI-subtyped to be LumA or Basal are shown. **Figure S4.** Youden analysis for optimal patient-level classification thresholds. Youden analysis was used to define the minimum percentage of patches that were subtyped as Basal (left) and Luminal A (right) to maximize agreement with RNA-seq-based classifications of Basal and Luminal A respectively. Shown here are TPR vs. FPR plots at various thresholds for the minimum percentage of patches subtyped as Basal (left) and Luminal A (right). The Youden index (i.e. the threshold value most distant from the x = y line) maximizes the TPR:FPR ratio. **Table S1.** Performance of PCA transformations. Variance captured at all three zoom-levels when increasing the number of dimensions in 256 principle component increments, starting at 256. Note that at 768 components, over 95% of variance is captured in all three zoom-levels. Euclidean distances between the original 2048-space vectors and PCA-estimated ones were computed and are reported here as an additional performance error metric. **Table S2.** Breast cancer k-means clusters. Descriptive statistics for 14 of the 24 different clusters identified in multiscale patch representations that were analyzed by a pathologist. The ten clusters not shown were excluded from further analysis due to having very little cellular content. A pathologist provided a binary label (cancer or non-cancer) for a total of 336 patches (24 randomly selected examples from each of the 14 clusters). Shown here is the number of patches in identified within each of these clusters, their relative representation of the total number of samples in this study (238,728), the average distance of any given patch to the cluster centroid (as a measure of scatter), and the percentage of patches inspected by a pathologist that contained cancer. **Table S3.** Basal vs. non-Basal patch-level train performance**. Table S4.** Balanced Basal vs. non-Basal classifier, patch-level train performance. **Table S5.** Basal vs. non-Basal classifier WSI-level performance on validation set.

## Data Availability

Training images and annotations are publicly available from TCGA sources.

## Abbreviations

BC, BRCA: Breast cancer
CAD: Computer-assisted diagnosis
CNN: Convolutional neural networks
CSCs: Cancer stem cells
DCIS: Benign ductal carcinoma in situ
ER (ERα/*ESR1*): Estrogen receptor (alpha)
FFPE: Formalin-fixed paraffin-embedded
FPR: False positive rate
H&E: Hematoxylin and eosin
HER2: Human epidermal growth factor receptor
HET: Heterogeneous
HR: Hazard ratio
IDC: Invasive ductal carcinomas
IHC: Immunohistochemistry
IMG: By image
IMS: Intrinsic molecular subtype
ISH: In situ hybridization
PAM50: Predictor Analysis of Microarray 50
PCA: Principal component analysis
PR (*PGR*): Progesterone receptor
RBF: Radial basis function
RNA-seq: RNA sequencing
SVM: Support vector machine
TCGA: The Cancer Genome Atlas
TMA: Tissue microarray
TPR: True positive rate
WSI: Whole-slide image
