# Supplementary material for: A deep learning image-based intrinsic molecular subtype classifier of breast tumors reveals tumor heterogeneity that may affect survival
Source: Breast Cancer Res. 2020 Jan 28;22:12. doi: 10.1186/s13058-020-1248-3 (PMC6988279; doi:10.1186/s13058-020-1248-3)
Supplement: Supplementary file 1 — Additional file 1: Figure S1. Identifying low-confidence PAM50 labels. PCA-plot showing clustering of patient samples using PAM50 genes. Subtype centroids are marked in dark circles, with lines to each patient assigned to those subtypes. Euclidean distance in this space was used to identify 104 patients that cluster significantly closer to a non-assigned centroid. Figure S2. Exemplary cancer-enriched multiscale patches. A total of 238,728 multiscale patches were clustered into 24 groups by k-means clustering and 336 representatives were selected for pathologist interpretation. Shown here are multiple zoom levels for the patches from the five most cancer-rich cluster centroids, as defined by pathologist inspection. Below each example patch are the group proportions and the percentage of patches containing cancer tissue. Figure S3. WSI-based IMS vs. RNA-seq-based PAM50 using test data only for unselected and low-confidence samples. WSI-based IMS vs. RNA-seq-based molecular PAM50 on test patients in Table 2 (unselected & low-confidence). a Kaplan-Meier curves for Luminal A and Basal-like based on molecular PAM50 calls with HR = 1.27 and log-rank tests P = 0.60. b Kaplan-Meier curves for Luminal A and Basal-like based on WSI IMS calls with HR = 1.66 and log-rank tests P = 0.11. In c, all the cases analyzed were molecularly classified as LumA, but the WSI-based system classified some of these as Basal (yellow); the expression levels of ESR1 and PGR for cases WSI-subtyped as either Basal or LumA (blue) are shown. d Similarly, the receptor levels of molecularly-subtyped Basal cases WSI-subtyped to be LumA or Basal are shown. Figure S4. Youden analysis for optimal patient-level classification thresholds. Youden analysis was used to define the minimum percentage of patches that were subtyped as Basal (left) and Luminal A (right) to maximize agreement with RNA-seq-based classifications of Basal and Luminal A respectively. Shown here are TPR vs. FPR plots at various thresholds [file 13058_2020_1248_MOESM1_ESM.docx]

**Additional file 1**

**Figure S1. Identifying low-confidence PAM50 labels**

**
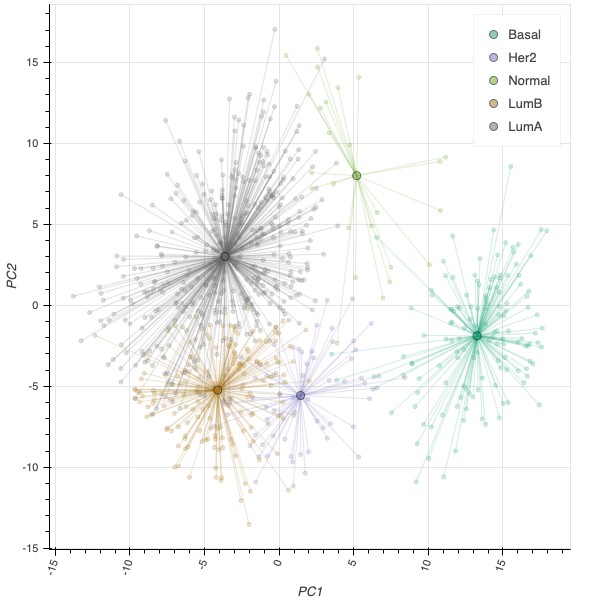
**

**Figure S1.** PCA-plot showing clustering of patient samples using PAM50 genes. Subtype centroids are marked in dark circles, with lines to each patient assigned to those subtypes. Euclidean distance in this space was used to identify 104 patients that cluster significantly closer to a non-assigned centroid.

**Figure S2. Exemplary cancer-enriched multiscale patches**

**
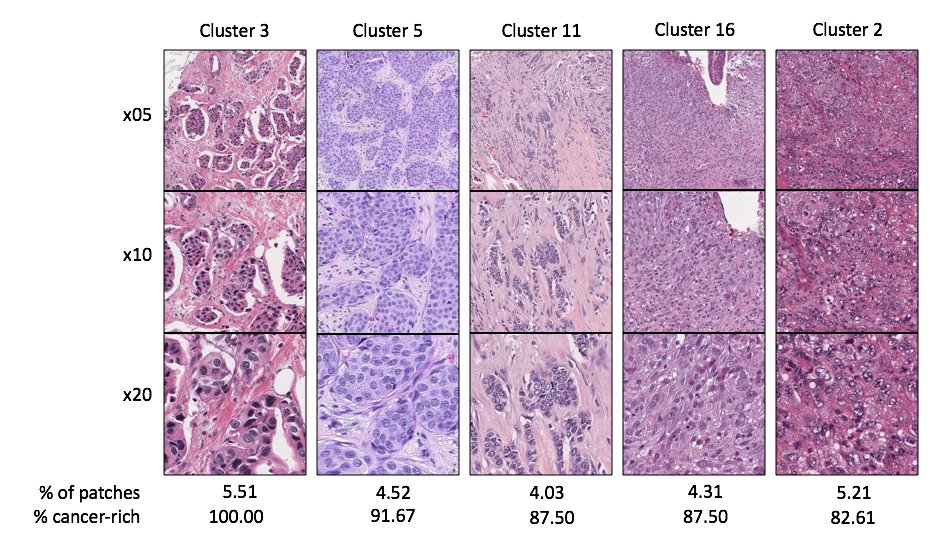
**

**Figure S2.** A total of 238,728 multiscale patches were clustered into 24 groups by *k*-means clustering and 336 representatives were selected for pathologist interpretation. Shown here are multiple zoom levels for the patches from the five most cancer-rich cluster centroids, as defined by pathologist inspection. Below each example patch are the group proportions and the percentage of patches containing cancer tissue.

**Figure S3. WSI-based IMS vs. RNA-Seq-based PAM50 using test data only for unselected and low-confidence samples.**

**
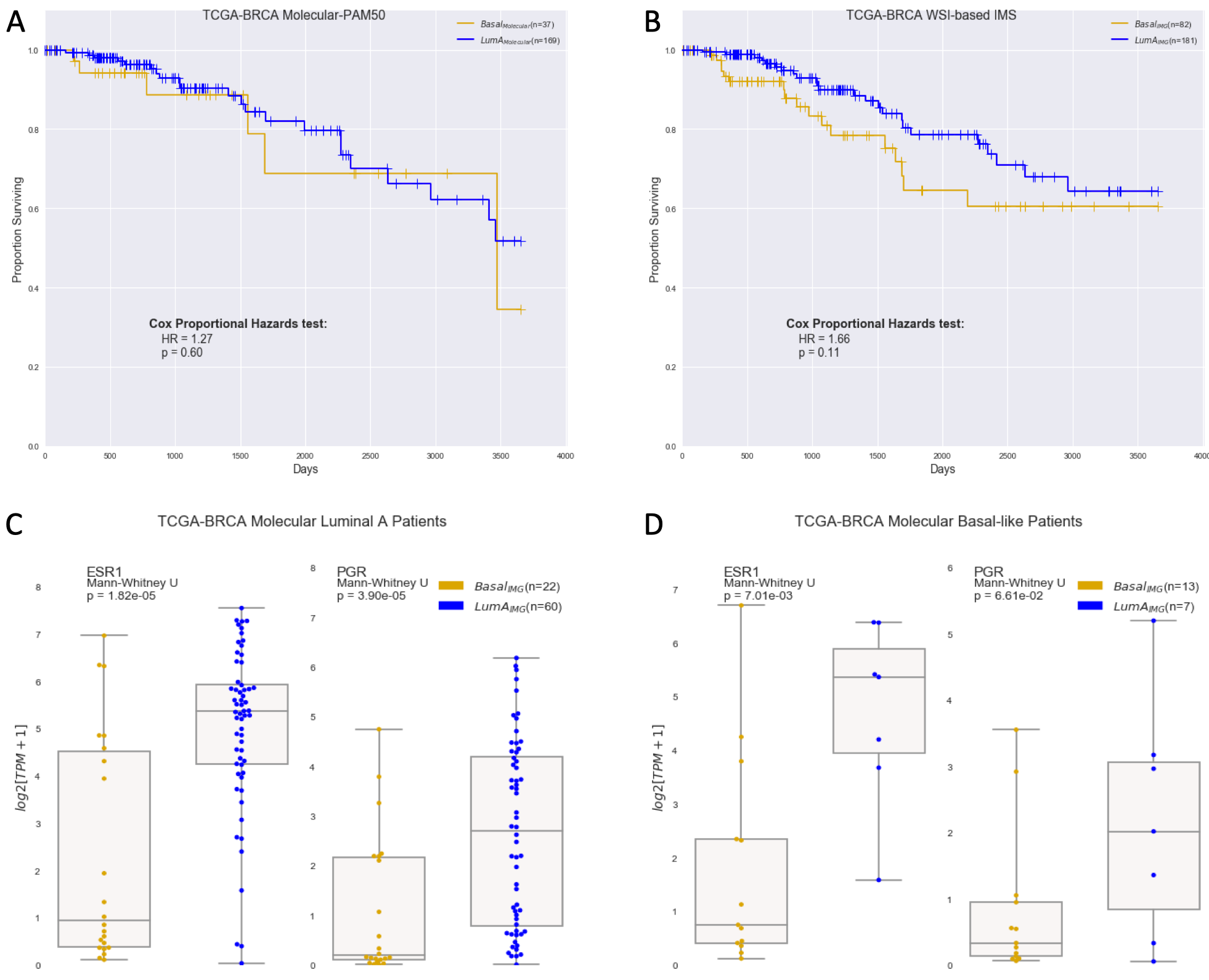
**

**Supplementary Fig. 3** WSI-based IMS vs. RNA-Seq-based molecular PAM50 on test patients in Table 2 (unselected & low-confidence). **a** Kaplan-Meier curves for Luminal A and Basal-like based on molecular PAM50 calls with HR = 1.27 and log-rank tests P = 0.60. **b** Kaplan-Meier curves for Luminal A and Basal-like based on WSI IMS calls with HR = 1.66 and log-rank tests P = 0.11. In **c**, all the cases analyzed were molecularly classified as LumA, but the WSI-based system classified some of these as Basal (yellow); the expression levels of ESR1 and PGR for cases WSI-subtyped as either Basal or LumA (blue) are shown. **d** Similarly, the receptor levels of molecularly-subtyped Basal cases WSI-subtyped to be LumA or Basal are shown.

**Figure S4. Youden analysis for optimal patient-level classification thresholds**

**
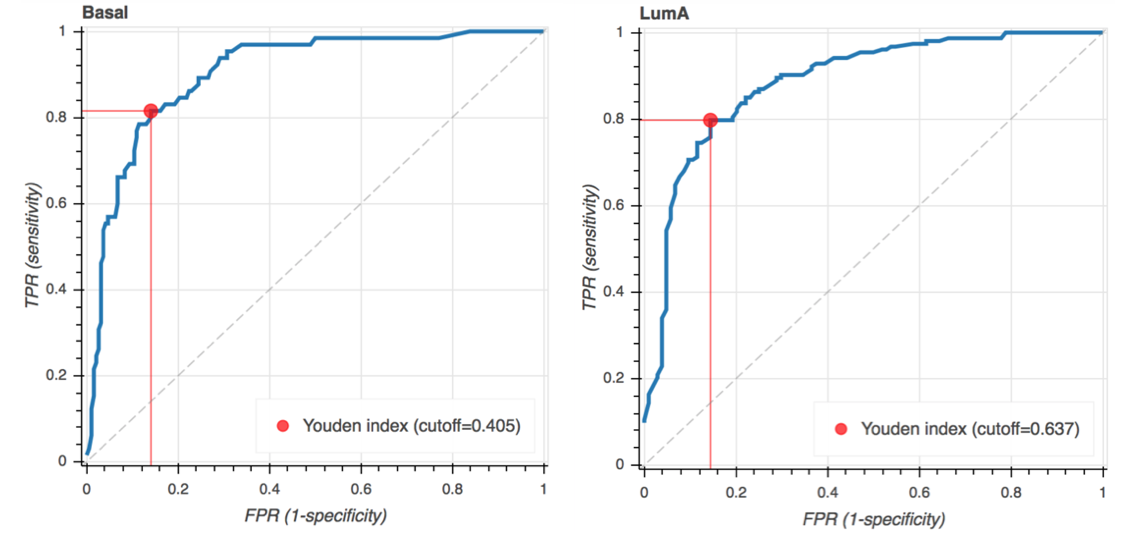
**

**Figure S4.** Youden analysis was used to define the minimum percentage of patches that were subtyped as Basal (left) and Luminal A (right) to maximize agreement with RNA-seq-based classifications of Basal and Luminal A respectively. Shown here are TPR vs. FPR plots at various thresholds for the minimum percentage of patches subtyped as Basal (left) and Luminal A (right). The Youden index (i.e. the threshold value most distant from the x=y line) maximizes the TPR:FPR ratio.

**Table S1. Performance of PCA transformations**

**Table S1.** Variance captured at all three zoom-levels when increasing the number of dimensions in 256 principle component increments, starting at 256. Note that at 768 components, over 95% of variance is captured in all three zoom-levels. Euclidean distances between the original 2048-space vectors and PCA-estimated ones were computed and are reported here as an additional performance error metric.

**Table S2. Breast cancer k-means clusters**

**Table S2.** Descriptive statistics for 14 of the 24 different clusters identified in multi-scale patch representations that were analyzed by a pathologist. The ten clusters not shown were excluded from further analysis due to having very little cellular content. A pathologist provided a binary label (cancer or non-cancer) for a total of 336 patches (24 randomly selected examples from each of the 14 clusters). Shown here is the number of patches in identified within each of these clusters, their relative representation of the total number of samples in this study (238,728), the average distance of any given patch to the cluster centroid (as a measure of scatter), and the percentage of patches inspected by a pathologist that contained cancer.

**Table S3. Basal vs. non-Basal patch-level train performance**

| **Linear SVM (C = 1.0) training using top 060 patches per WSI from 582 train WSIs** | **34,745** |
| --- | --- |
| % Basal | 15.8440 |
| % Non-Basal | 84.1560 |
| Patch-level training sensitivity | 64.7230 |
| Patch-level training specificity | 95.4446 |
| **Patch-level training accuracy** | **90.5771** |

**Table S4. Balanced Basal vs. non-Basal classifier, patch-level train performance**

| **Linear SVM (C = 1.0) training using top 060 patches per WSI from 582 train WSIs** | **11,011** |
| --- | --- |
| **% Basal** | **49.9955** |
| **% Non-Basal** | **50.0045** |
| Patch-level training sensitivity | 89.4278 |
| Patch-level training specificity | 89.7203 |
| **Patch-level training accuracy** | **89.5741** |

**Table S5. Basal vs. non-Basal** **classifier WSI-level performance on validation set**

| **Classifier** | **ROC AUC** | **Threshold =**  **% Basal patches in training set** | **Accuracy** | **Sensitivity** | **Specificity** |
| --- | --- | --- | --- | --- | --- |
| **Basal vs. non-Basal** **classifier** | **0.8259** | 0.1584 | **86.82** | 38.64 | 96.73 |
| **Balanced Basal vs. non-Basal** **classifier** | **0.8607** | 0.5000 | **87.21** | 68.18 | 91.12 |
